# An Intervention to Promote Healthy Weight: Nutrition and Physical Activity Self-Assessment for Child Care (NAP SACC) Theory and Design

**Published:** 2007-06-15

**Authors:** Sara E Benjamin, Alice S Ammerman, Dianne S Ward, Sarah C Ball, Janice K Sommers, Meg Molloy, Janice M. Dodds

**Affiliations:** Department of Ambulatory Care and Prevention, Harvard Medical School and Harvard Pilgrim Health Care; Department of Nutrition, Schools of Public Health and Medicine, University of North Carolina at Chapel Hill, Chapel Hill, NC; Department of Nutrition, Schools of Public Health and Medicine, University of North Carolina at Chapel Hill, Chapel Hill, NC; Center for Health Promotion and Disease Prevention, University of North Carolina at Chapel Hill, Chapel Hill, NC; Center for Health Promotion and Disease Prevention, University of North Carolina at Chapel Hill, Chapel Hill, NC; North Carolina Prevention Partners, Chapel Hill, NC; Departments of Nutrition and Maternal and Child Health, Schools of Public Health and Medicine, University of North Carolina at Chapel Hill, Chapel Hill, NC

## Abstract

Health professionals are faced with the growing challenge of addressing childhood overweight. Few overweight prevention efforts have targeted young children, particularly children in child care settings. We describe the theory and development of a novel nutrition and physical activity environmental intervention. On the basis of findings from interviews and focus groups, a review of national recommendations and standards, and a review of the literature, we developed a nutrition and physical activity environmental self-assessment instrument to assess physical activity and nutrition policies and practices in child care settings. An intervention model was built around existing public health infrastructure to support use of the self-assessment instrument and encourage environmental changes at the child care level, and this intervention model became the Nutrition and Physical Activity Self-Assessment for Child Care (NAP SACC) program. The NAP SACC program was designed for dissemination and has potential for implementation in many settings. Broad interest in NAP SACC has been expressed by a number of states and institutions, and many groups are using NAP SACC intervention and materials. The NAP SACC program shows promise as a useful approach to promoting healthy weight behaviors in child care settings.

## Introduction

Childhood overweight has become an epidemic in the United States with approximately 33.6% of children described as overweight or at risk of overweight (1). Among children aged 2 to 5 years, approximately 26.2% are now overweight or at risk of overweight, nearly triple the rate during the early 1970s (1,2). These statistics emphasize the need for more programs addressing overweight prevention for children aged 2 to 5 years. Past efforts have focused on individual level interventions (2-10) or single aspects of overweight prevention, such as reduced television viewing (11-14). Individual-level efforts alone are not sufficient to address this public health problem (15,16).

Broader approaches to address healthy weight in children are needed, the advantage being the ability to create a nutrition and physical activity environment that supports healthful behavioral choices (15). Although several such interventions exist, only a handful have attempted to reach young children (5,11,17-19).

Targeting young children in child care is important because of the large number of children now in out-of-home child care. During the past several decades, the number of dual-income families has increased markedly, which has led to more children spending much of their days in a child care setting (20-22). According to the 2001 National Household Education Survey, 74% of all children aged 3 to 6 years are in some form of nonparental care, and 56% are in a center-based child care program (23). Consequently, many children consume 50% to 100% of their Recommended Dietary Allowances in child care settings (24), and early development of nutrition attitudes and behaviors are subject to influence by nonfamily members. In addition, children must be given opportunities to be physically active in child care, since many children are spending a large proportion of their time in child care (21,22). Recent physical activity guidelines for preschoolers recommend at least 60 minutes and up to several hours of unstructured physical activity and at least 60 minutes of structured physical activity daily (25). Given the amount and proportion of time many children spend in child care each day, opportunities for active play must be included in the child care experience. Because of these factors, the child care environment lends itself to healthy weight interventions.

Currently, the child care environment is largely evaluated for safety and compliance. Most licensed child care facilities are governed by state laws that may or may not include regulations addressing healthy eating and physical activity (26). In child care facilities that participate in the Child and Adult Care Food Program (CACFP) (27), the federal food assistance program that provides reimbursement for meals and snacks, providers are required to comply with more specific guidelines specifying meal components and amounts of food served; however, participation in this program is voluntary (except for Head Start program participants) and limited to facilities that serve 25% or more low-income children. Although CACFP provides portion size requirements for meals and snacks, it does not include any nutrient-based standards for foods served. Few, if any, regulations exist for physical activity at child care centers. Child care facilities that would like to improve and enhance their nutrition and physical activity environments must do so through self-initiated or voluntary efforts.

This paper describes the development of an environmental intervention to address healthy weight for children in child care in North Carolina. The Nutrition and Physical Activity Self-Assessment for Child Care (NAP SACC) program was developed in the winter of 2001 and spring of 2002 as an environmental intervention for child care centers that could be sustained over time and easily disseminated within an existing public health infrastructure. The goal of the NAP SACC intervention is to improve the diet and physical activity environment at child care centers in support of healthy weight in children, to contribute to the marketability of the child care center, and to provide child care staff with continuing education in child nutrition and physical activity practices.

## Background

Many preliminary activities were undertaken to develop NAP SACC, including conducting formative research, examining existing standards and policies, reviewing research evidence about nutrition and physical activity at child care, and forming a local advisory group.

### Formative research

The purpose of the formative research was threefold: 1) to gain insight into child care provider and parent views of the nutrition and physical activity environments in North Carolina (NC) child care centers, 2) to understand how the regulatory and rating systems influence nutrition and physical activity in the child care settings, and 3) to identify opportunities to enhance and promote positive nutrition and physical activity behaviors in children in child care settings through environmental change. A preliminary literature review was conducted to document the science base surrounding physical activity and nutrition within the child care setting. State and national expert groups working in the child care nutrition and physical activity arena, including the North Carolina Partnership for Children and the National Training Institute for Child Care Health Consultants (a grant-funded center founded to develop a national training program for health consultants to child care facilities), were then contacted to gain insight into unpublished, but current, approaches and views in this area. These initial steps guided the development of two qualitative instruments: a key informant interview guide for child care personnel and a focus group guide for parents of children in child care.

Fifteen key informant interviews with child care providers, including directors, assistant directors, teachers, and food service staff, were conducted. Three parent focus groups, involving 17 parents of young children in child care, were held. Interviews and focus groups were planned to ensure diversity among participants with regard to demographic characteristics. Parents from diverse racial and ethnic backgrounds were recruited to participate in the formative data collection. Parents of children with special needs and parents of children attending both full-time and part-time care were included in the interviews and focus groups. Trained interviewers conducted the key informant interviews in person or over the phone, and experienced facilitators conducted the focus groups at a convenient location. Focus group participants were offered child care and compensation for participation, with assurances that their comments would remain confidential. All interviews and focus groups were tape recorded and transcribed with additional notes taken during both. A complete transcription of each interview and focus group was then created using the notes and tape recordings. A coding method developed by study investigators identified themes used to organize and synthesize the qualitative data.

Important findings from both the interviews and focus groups included three major themes: 1) views on physical activity in the child care setting, 2) views on nutrition in the child care setting, and 3) perception of NC regulatory and rating systems. Overall, child care providers reported believing that adequate opportunities for physical activity exist and that few barriers to creating new opportunities exist. Although providers reported they limited children's use of television, the definition of "limited" was widely interpreted. Parents reported believing that their children are active all day in child care, far more than their school-aged children; however, they expressed concern about the relative safety of physical activity and noted time and safety as barriers to active play at home.

Most providers felt that the meals served in their centers were balanced and healthy, with family-style meal service the norm after the age of 3 years. However, parents had varied opinions; some parents thought that meals lacked variety, and other parents thought meals were healthy. Both providers and parents felt that the child care meal environment, including proper role modeling by staff, is important. Interestingly, no providers listed cost as a barrier to serving healthy foods.

Overall, child care providers reported using CACFP rules to guide menu development and meal preparation. Providers also reported that use of a NAP SACC rating scale might offer credibility among parents considering child care options. Most parents, although interested in the newly implemented five-star rating system, reported giving more credence to their personal impressions when choosing a child care facility.

### Review of current recommendations, standards, and research evidence

We conducted a thorough review of nutrition and physical activity standards and recommendations related to child care and to children aged 2 to 5 years. Included in the search were regulations and performance standards from *Caring for Our Children: National Health and Safety Performance Standards: Guidelines for Out-of-Home Child Care* (28,29), the Head Start program (30), the National Association for the Education of Young Children (NAEYC) (31), and the Special Supplemental Nutrition Program for Women, Infants, and Children (WIC) (32). For guidance on the development of the physical activity component of NAP SACC, the National Association for Sport and Physical Education's (NASPE's) *Active Start: A Statement of Physical Activity Guidelines for Children Birth to Five Years* (25) was used. Recommendations from the Early Childhood Environment Rating Scale (ECERS) (33) and the Infant and Toddler Environment Rating Scale (ITERS) (34), created by the FPG Child Development Institute at the University of North Carolina at Chapel Hill, also provided valuable information.

In addition to reviewing recommendations and standards, we reviewed the scientific literature addressing the nutrition and physical activity behaviors of young children. Areas in which clear recommendations and standards did not exist were emphasized. Results of the standards and research review are presented later in this paper.

### Formation of the advisory group

An advisory group of child health professionals (a child nutritionist and two Child Care Health Consultants), child care center staff (teachers, directors, and food preparers), and a county extension agent was formed and has convened yearly since 2001. Their role was to guide development of the intervention process and materials by providing insight on the appropriateness and usability of the intervention and materials with the target audience. The advisory group provided regular feedback through mail and e-mail and, in addition to reviewing program materials, helped shape the overall direction and approach of the intervention as the program progressed to the implementation stage.

## Intervention Design and Development

The NAP SACC program contains a number of components, including a self-assessment instrument, continuing education workshops, collaborative action planning and technical assistance materials, and an extensive resource manual that includes copy-ready materials. Development of each of these components, including the conceptual model, is described below.

### Conceptual model

Influencing the nutrition and physical activity behaviors of children necessitates an intervention approach that considers individual behaviors as well as the environments in which behaviors take place. Physical activity is heavily affected by the social environment, where children can learn behaviors through observing the teacher or other adults in the center. Similarly, dietary choices are influenced by the physical environment through food availability and adult role modeling. This inherent relationship between environments and behaviors, coupled with strong support from intervention research, suggested the utility of using Social Cognitive Theory (SCT) as the theoretical basis for the NAP SACC nutrition and physical activity environmental intervention (35). SCT identifies several crucial factors that influence behavior change, including expectancies, observational learning, self-efficacy, behavioral capability, environment, situation, reinforcement, and reciprocal determinism. The NAP SACC intervention was designed to reflect these key constructs.

### Intervention steps

The NAP SACC intervention was designed for implementation through an existing infrastructure of public health professionals, typically registered nurses and health educators who are trained as NAP SACC consultants. Key steps in the intervention included the following:

- Child care center directors and related staff complete the self-assessment instrument to assess center nutrition and physical activity policies, practices, and overall environment.
- NAP SACC consultants work with centers to develop an action plan to improve at least three target areas of concern identified from the self-assessment instrument. Center directors select their areas of interest to facilitate the most fitting and lasting environmental changes.
- NAP SACC consultants deliver to center staff three NAP SACC continuing education workshops on 1) childhood overweight, 2) healthy eating for children, and 3) physical activity for children.
- NAP SACC consultants provide ongoing targeted technical assistance, through in-person visits and telephone follow-up, to support implementation of planned policy, practice, and environmental changes.
- Centers use a follow-up self-assessment instrument to evaluate changes made to the center during the 6-month intervention period.

### Development of the self-assessment instrument and workshops

Although other instruments to improve child care quality (e.g., ECERS and ITERS rating scales) provided some guidance when developing this instrument, we decided to take a self-assessment approach to improving child care environments, similar to that of the School Health Index (36). Our formative work suggested that a self-assessment approach would help target areas for attention and provide more sustainable improvements through voluntary participation and self-initiated change. Center-directed assessment allows child care settings to evaluate their nutrition and physical activity environments without repercussion from regulatory or licensing groups. Thus, no outside rater is needed. In addition, completion of the self-assessment instrument is quick and easy. The instrument is designed to allow the child care center director to answer questions, with assistance from key center staff (e.g., lead teacher, cook), about the center environment.

The initial instrument included 44 questions from nine nutrition and six physical activity areas that had either a demonstrated (evidence-based) or perceived (expert-based) relationship to childhood overweight based on the review of standards and research findings. Key areas, along with references from standards, recommendations, and research are presented in Table 1. Each of the 44 questions had three possible response categories, with 1, 2, or 3 points assigned for each response (1 = minimum standard, 2 = good, 3 = best practice). The total score range for the instrument was 44 to 132 points.

Once the instrument was developed, it was reviewed by eight NC experts in the fields of child development, child care, nutrition for young children, and physical activity for young children. These experts included physicians, registered dietitians, and physical activity and child development researchers. A number of improvements were suggested, and changes were incorporated. In addition, the advisory group evaluated the self-assessment instrument before conducting the pilot study.

As the program moved from the development to implementation stage, the advisory group provided a number of suggestions that led to crucial modifications to the intervention. The three workshops, which were at first designed to provide training to interested child care center staff, became a more prominent step in the intervention. The advisory group suggested we seek state licensing agency approval for continuing education (CE) credits for each of the workshops to help ensure greater participation from center staff and to increase support for the intervention. We subsequently learned that obtaining adequate CE credits is a significant challenge for many center staff, and providing credits for NAP SACC workshops was a substantial incentive for workshop participants.

### Support materials

On the basis of formative data and guidance from our advisory group, we developed a tool kit of technical assistance and background materials to facilitate effective intervention implementation by the NAP SACC consultants. The NAP SACC tool kit was designed by local health professionals and researchers from the University of North Carolina. It consists of several components designed to provide the consultant with additional materials and handouts for use during the center workshops and the general intervention process. The tool kit consists of the NAP SACC notebook, copies of educational materials (paper and CD-ROM) for the three NAP SACC workshops, and handouts for centers and parents linked to each of the 15 key areas in NAP SACC.

Although the NAP SACC consultants were thoroughly trained by research staff to implement the NAP SACC intervention, we felt that they may have additional questions on nutrition and physical activity extending beyond the scope of the training. This was the rationale behind the development of the NAP SACC resource manual. The resource manual is divided into four sections: 1) self-assessment, 2) nutrition, 3) physical activity, and 4) resources. Both the nutrition and physical activity sections are further divided according to the NAP SACC key areas and include a rationale for each best practice along with potential challenges for implementing the practice and tips to help child care providers address these challenges. Table 2 depicts a sample best practice guideline along with likely implementation challenges and tips to circumvent them. With this manual, NAP SACC consultants have technical assistance materials to support the center in implementing the intervention.

The resource section of the NAP SACC manual was compiled from information on child nutrition, meal preparation, outdoor activity and safety, indoor activity and safety, and physical activity. It was designed to give NAP SACC consultants and centers ideas for improvement and to provide additional background knowledge relative to child nutrition and physical activity. Additionally, it provides a broad selection of recipes for center use.

Workshop materials are provided as both paper copies and PowerPoint presentations on a CD-ROM in order to accommodate the needs of the NAP SACC consultants. A separate folder for each workshop is included in the tool kit with everything needed to conduct the workshops at the child care centers. The center handouts were created from the NAP SACC resource manual and emphasize best practices, tips, and suggestions for working with parents. A handout for each NAP SACC key area provides additional guidance for child care staff.

### Pilot testing and revision

The NAP SACC program was pilot tested for feasibility and acceptability in a convenience sample of 19 child care centers from eight counties in NC. The program was delivered by Child Care Health Consultants, who volunteered to be trained as NAP SACC consultants for this pilot. Results from the pilot study are reported elsewhere (37).

On the basis of feedback from the pilot study, additional revisions were made to the self-assessment instrument. A number of questions were reworded to improve clarity, and some of the response categories were modified to reflect typical practice. Upon completion of this process, the self-assessment instrument was once again sent out for expert review. Ten national experts in the fields of child development, child care, nutrition for young children, and physical activity for young children were asked to review the instrument. As a result, the response categories were expanded from three to four categories, 11 questions were added to the instrument, and one question was removed. Reliability and validity testing of the self-assessment instrument is under way in a sample of child care centers.

## Discussion and Future Directions

To our knowledge, NAP SACC is the first intervention to address healthy weight at the child care center with a focus on both the nutrition and physical activity social and physical environment and on staff education. Using extensive formative data and guidance from an advisory group of child care experts and practitioners, NAP SACC was developed to be an evidence- and theory-based intervention that is guided by a self-assessment completed by the child care center director and relevant staff (e.g., cook, lead teacher, assistant director). Technical assistance and support for change are provided by NAP SACC consultants, individuals already working in local communities who receive supplemental training and support materials to expand their role to include nutrition and physical activity. NAP SACC has been pilot tested for feasibility and usability and found to be appropriate for the child care center setting and shows promise as an effective intervention. Pilot testing was conducted using Child Care Health Consultants to deliver the NAP SACC intervention, but many other health professionals could be employed. NAP SACC consultants were able to incorporate the program into their existing professional duties and were not paid as research staff. By using an existing public health professional, the NAP SACC program shows promise of being a sustainable and easy-to-implement intervention.

The problem of overweight in preschool age children is a serious one, with 13.9% of children aged 2 to 5 years classified as overweight according to recent National Health and Nutrition Examination Survey statistics (1). With more children spending increased time in child care (23), the child care setting is ideal for healthy weight intervention. A few curricular and educational programs have been developed for use in child care, which have resulted in some promising behavioral changes in the areas of saturated fat intake (19) and TV viewing (11), with one study demonstrating an impact on body mass index (BMI) (less increase than controls) (17). However, to our knowledge there have been no published studies of environmental- and policy-level interventions in child care that target the provider. We believe that sustainable improvements in nutrition and physical activity can best be facilitated through more upstream interventions that address the food and physical activity environments. This includes not only the available play space and equipment and foods served for meals and snacks but also role modeling by child care staff and center policies affecting everything from outdoor playtime in bad weather to snacks brought in by parents for birthday celebrations.

Several improvements to the NAP SACC intervention and supporting materials have been made based on feedback from the pilot intervention and advisory group. The self-assessment instrument has been expanded to include four, instead of three, response categories. Center director feedback suggested that directors gravitated toward the middle response and wished to have just one more category of choice. Additionally, staff feedback suggested that they truly enjoyed the CE credit workshops but wished to learn more about their own health and prevention of obesity. Thus, a Personal Health and Wellness workshop was added to the program.

In addition to the feasibility pilot study (37), several other research efforts are under way to more rigorously evaluate the behavioral and physiologic impact of NAP SACC on children within participating centers. Currently, the NAP SACC intervention is being evaluated in 33 counties and 96 child care centers in NC. Environmental improvements at the intervention centers, as documented by a researcher-administered observation and assessment system, are being compared with improvements at control child care centers receiving a delayed intervention. Child-level data (dietary intake and physical activity) will also be collected and assessed. In addition, minimal standards for implementation of the intervention (a dose-response analysis) will be assessed to determine the amount, or dose, of intervention required to elicit environmental change at the child care center. Also being tested is a more intensive NAP SACC intervention (12 months instead of 6 months) to measure the intervention's effect on blunting the rise in BMI that occurs in this age group. Additionally, a family-based component of NAP SACC, designed to reinforce the policy and environmental changes within the centers, is under way.

Because the NAP SACC program has been designed for dissemination since its inception, we believe it has potential for implementation in many different types of child care settings (e.g., preschools, child care centers, family child care homes) where it can contribute to facilitating healthier weight among preschool children. Broad interest in NAP SACC has been demonstrated by a number of institutions and public health agencies, and many states are using the revised NAP SACC intervention and materials. The NAP SACC program shows promise as a useful approach to promote healthy weight in child care settings around the country. 

## Figures and Tables

**Table 1:** Key Areas on the NAP SACC Self-Assessment Instrument, North Carolina, 2001–2006

Area	**Description (References** a **)**
Nutrition 1	Fruits and Vegetables (1-10)
Nutrition 2	Fried Foods and High Fat Meats (2,3,6,11-14)
Nutrition 3	Beverages (1,2,7,10,13-19)
Nutrition 4	Menus and Variety (1-3,6,14,20,21)
Nutrition 5	Meals and Snacks (1,2,6,14,22-30)
Nutrition 6	Foods Outside of Regular Meals and Snacks (1-3,14,21,29)
Nutrition 7	Supporting Healthy Eating (1,2,6,7,14,31,32)
Nutrition 8	Nutrition Education for Children, Parents, and Staff (1,2,6,14,21,29,33,34)
Nutrition 9	Nutrition Policy (1,10,18,21)
Physical Activity 1	Active Play and Inactive Time (3,10,29,35-46)
Physical Activity 2	TV Use and TV Viewing (7,10,47-49)
Physical Activity 3	Play Environment (37,39,40,46,50,51)
Physical Activity 4	Supporting Physical Activity (37,39,40,46,52,53)
Physical Activity 5	Physical Activity Education for Children, Parents, and Staff (29,35,37,38,40,46,54)
Physical Activity 6	Physical Activity Policy (35,40,46,55)

NAP SACC indicates Nutrition and Physical Activity Self-Assessment for Child Care.

a See Appendix for references for this table.

**Table 2:** NAP SACC Resource Manual, Sample Best Practice Guideline, North Carolina, 2001–2006

**Juice is served as a fruit/vegetable serving once per week or less**	
**Rationale**	
Fruits and vegetables provide more nutrition than fruit juice for children. Children tend to fill up on juice and not eat much at the meal or snack. Excess juice consumption has been linked to promotion of overweight among children. Fruit juice contains natural sugars that may adhere to teeth and cause cavities. Children benefit more from consuming fruits and vegetables during snacks and meals rather than fruit juice.	
**Challenges**	**Tips**
Fruit juice is inexpensive and easy to prepare.	Help staff identify easy-to-eat alternatives to juice like orange sections, fruit salad, or banana halves. Juice can still be offered and does not need to be banned. However, easy and healthful alternatives should be offered every day.
Children enjoy drinking fruit juice and ask for it when they are thirsty.	Children do enjoy juice and often ask for it when they are thirsty. If a child has already had juice, the staff should offer water instead.
Staff and parents believe that fruit juice is healthy and encourage children to drink it.	If a child drinks juice instead of water, the juice may depress the child's appetite for whole foods, provide more calories than needed, cause diarrhea, and expose the child's teeth to excess sugar. Remind staff of the benefits and limitations of juice in a child's diet.

NAP SACC indicates Nutrition and Physical Activity Self-Assessment for Child Care.
